# Top-Down Regulation, Climate and Multi-Decadal Changes in Coastal Zoobenthos Communities in Two Baltic Sea Areas

**DOI:** 10.1371/journal.pone.0064767

**Published:** 2013-05-24

**Authors:** Jens Olsson, Lena Bergström, Anna Gårdmark

**Affiliations:** Institute of Coastal Research, Department of Aquatic Resources, Swedish University of Agricultural Sciences, Öregrund, Sweden; University of Florida, United States of America

## Abstract

The structure of many marine ecosystems has changed substantially during recent decades, as a result of overexploitation, climate change and eutrophication. Despite of the apparent ecological and economical importance of coastal areas and communities, this aspect has received relatively little attention in coastal systems. Here we assess the temporal development of zoobenthos communities in two areas on the Swedish Baltic Sea coast during 30 years, and relate their development to changes in climate, eutrophication and top-down regulation from fish. Both communities show substantial structural changes, with a decrease in marine polychaetes and species sensitive to increased water temperatures. Concurrently, opportunistic species tolerant to environmental perturbation have increased in abundance. Species composition show a similar temporal development in both communities and significant changes in species composition occurred in both data sets in the late 1980s and early 1990s. The change in species composition was associated with large scale changes in climate (salinity and water temperature) and to the structure of the local fish community, whereas we found no effects of nutrient loading or ambient nutrient concentrations. Our results suggest that these coastal zoobenthos communities have gone through substantial structural changes over the last 30 years, resulting in communities of different species composition with potentially different ecological functions. We hence suggest that the temporal development of coastal zoobenthos communities should be assessed in light of prevailing climatic conditions considering the potential for top-down effects exerted by local fish communities.

## Introduction

The ecosystems of the Baltic Sea have as many other marine systems worldwide, gone through substantial structural change during recent decades [1], [2]. This has been manifested as trophic cascades, caused by a decrease in top predators and a subsequent drop in the degree of top-down regulation [3]. For example, the collapse of the Eastern Baltic cod (*Gadus morhua*) stock during the late 1980s has been shown to affect the pelagic food-chain, down to zooplankton and phytoplankton both in the central Baltic Sea [3] and in nearby ecosystems [4]. The cod decline was likely the result of overexploitation and unfavorable climatic conditions [1], in that the Baltic Sea has become warmer and less saline during recent decades. These environmental changes have also been associated with substantial and synchronous structural changes in coastal fish communities in the Baltic Sea [5], involving a decrease in marine piscivores (cod), an increase in some piscivores of freshwater origin (perch, *Perca fluviatilis*), and concurrent decreases in some freshwater benthivores (e.g. roach, *Rutilus rutilus*). However, the potential links between observed large-scale changes in offshore and coastal fish communities and zoobenthic communities have not been thoroughly investigated.

The function and structure of coastal zoobenthos communities are suggested to be mainly governed by local conditions [6]–[8], generally being highly sensitive to low oxygen conditions and other eutrophication related effects, reviewed in [9]. In addition, hydrographical factors, such as water temperature and salinity levels as well as freshwater run-off, and sediment quality and structure have been identified as of importance in explaining variation in zoobenthos community structure [6], [8], [10]–[12]. With respect to the Baltic Sea, the long-term development of a coastal zoobenthos community in the Gulf of Finland was attributable to mainly increasing water temperatures and decreasing salinity levels [13]. The effects of top-down regulation and potential links between zoobenthos community structure and changes at higher trophic levels have not been as thoroughly explored, but might also have a significant impact on the structuring of coastal ecosystems and zoobenthos communities [14]–[21]. To date, however, we have limited knowledge on the integrated effects of these environmental factors for the long-term development of zoobenthos communities and the relative contribution of local and large scale processes, especially in coastal areas. It is also unknown whether the long-term development zoobenthos communities are consistent with that of higher trophic levels or not.

Strong bottom-up effects could mediate concurrent changes in taxa across trophic levels favoured by similar environmental conditions, whereas strong top-down effects might cause cascading effects trough the food-web, ultimately affecting ecosystem structure, function and resilence [15], [16], [18], [20]. Low densities of coastal predatory fish in the coastal zone of the Baltic Sea have, for example, been suggested to favour a predatory release on meso-predatory fish (i.e. sticklebacks). Increased densities of meso-predatory fish have in turn decreased the abundances of grazing macro zoobenthos species resulting in blooms ephemeral filamentous algae [16], effects that are similar to those resulting from eutrophication in shallow coastal areas.

In this study we assess the temporal development of zoobenthos communities from two Baltic Sea coastal areas, one in the eastern Baltic Proper and one in the south-eastern Bothnian Sea, during the last 30 years in relation to changes in ambient environmental factors and the structure of local fish communities. We address if there are common patterns in the development of species composition in both areas, and to what extent changes in species composition could be associated with the concurrent development in variables reflecting climate, eutrophication and local fish community structure. In this paper, we demonstrate a similar development of the species composition in both communities assessed, and that a significant part of the variation in species composition is a associated with decreasing salinity levels, increasing water temperatures and a concurrent change in local fish community composition.

## Materials and Methods

### Community data

**Figure 1:**
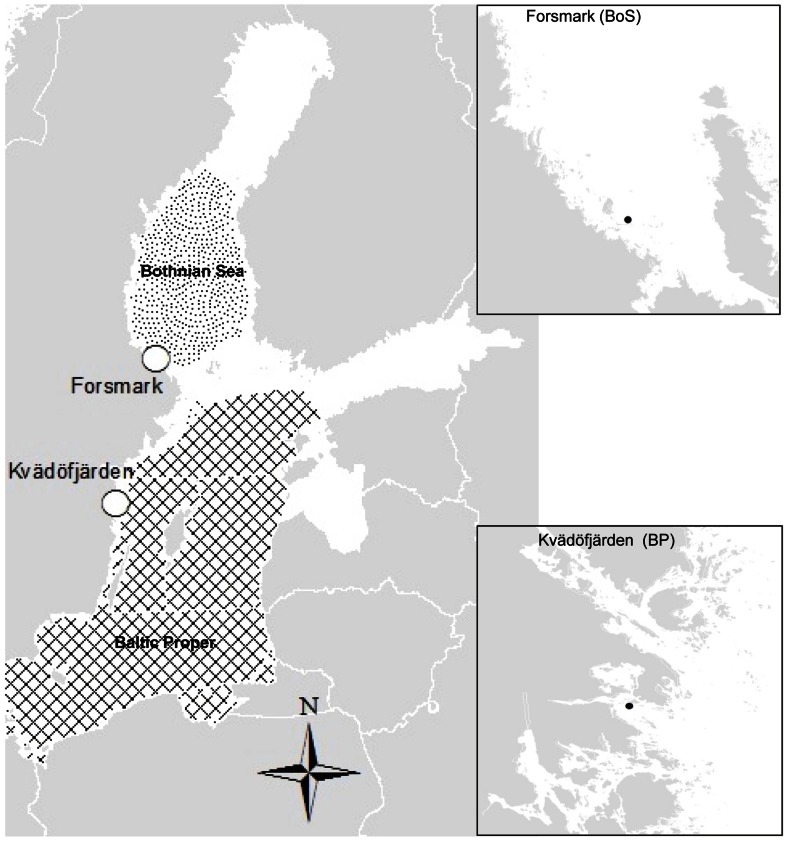
Location of the two areas for zoobenthos sampling. Kvädöfjärden (Baltic Proper, BP) and Forsmark (Bothnian Sea, BoS).

We analysed species abundance data (individuals/m^2^) from two coastal areas of the Baltic Sea, Kvädöfjärden (eastern Baltic Proper) and Forsmark (south-eastern Bothnian Sea; Figure 1). Data covered 1976–2008 (Kvädöfjärden) and 1980–2008 (Forsmark). In each area, five samples were collected annually at a depth of 22–24 meters in Kvädöfjärden and 16 meters in Forsmark. Sampling was performed on soft substrates bottoms in spring (May) using a van Veen grab sampler, according to the standards of [22]. Samples were sieved *in situ* through a 0.5 mm mesh and preserved in formalin prior to further analyses at the lab, where all organisms were identified to the nearest possible taxon. In 1980, 1982 and 1983 samples Forsmark were taken using an Ekman grabber. This did, however, only have a minor effect on the overall development of the species in the area (unpublished data), and thus on the outcome of the analyses. Monitoring has been performed by the same institute (Institute of Coastal Research) over all years.

There were some differences in species composition between the two data sets, but bivalves, amphipods and polychaetes was of dominating abundance in both communities (Table 1). Other common taxa included gastropods, isopods and insects (*Chironomidae*). In total, 16 species were recorded in Kvädöfjärden and 15 in Forsmark data set, representing both marine and freshwater taxa (Table 1). The numbers probably underestimate true species richness in each area, as they only include species represented in the applied monitoring method. We therefore restricted our analyses to assess relative changes in community compositions over time in each area, and in the text, the term “community” will refer to the sampled part of the community.

**Table 1:** Occurrence of the species included in the data sets assessed in the study, Kvädöfjärden (BP) and Forsmark (BoS).

Scientific name	Abbreviation^1^	Taxonomic class	Average abundance^2^	Data set
*Bathyporeia pilosus*	B pilosus	*Amphipoda*	17.9	Forsmark
*Bylgides sarsi*	B sarsi	*Polychaeta*	13.5	Kvädöfjärden
*Chironomidae sp*	–	*Diptera*	18.4, 1.7	Kvädöfjärden, Forsmark
*Corophium volutator*	C volutator	*Amphipoda*	0.2, 45.2	Kvädöfjärden, Forsmark
*Halicryptus spinulosus*	H spinolosus	*Priapulidae*	4.7	Kvädöfjärden
*Hydrobiidae sp*	–	*Gastropoda*	0.8	Kvädöfjärden
*Gammarus sp*	–	*Amphipoda*	0.8	Forsmark
*Macoma balthica*	M balthica	*Bivalvia*	430.6, 468.7	Kvädöfjärden, Forsmark
*Manayunkia aestuarina*	M aestuarina	*Polychaeta*	0.42	Forsmark
*Marenzelleria sp*	–	*Polychaeta*	37.9, 205.1	Kvädöfjärden, Forsmark
*Monoporeia affinis*	M affinis	*Amphipoda*	543.7, 43.2	Kvädöfjärden, Forsmark
*Mytilus edulis*	M edulis	*Bivalvia*	0.5	Kvädöfjärden
*Neomysis integer*	N integer	*Malacostraca*	0.3	Forsmark
*Nereis diversicolor*	N diversicolor	*Polychaeta*	0.3, 0.7	Kvädöfjärden, Forsmark
*Oligochaeta sp*	–	*Oligochaeta*	0.8, 129.0	Kvädöfjärden, Forsmark
*Potamopyrgus antipodarum*	P antipodarum	*Gastropoda*	4.4, 45.3	Kvädöfjärden, Forsmark
*Prostoma obscurum*	P obscurum	*Enopla*	0.4	Kvädöfjärden
*Pygospio elegans*	P elegans	*Polychaeta*	0.2, 14.1	Kvädöfjärden, Forsmark
*Saduria entomon*	S entomon	*Isopoda*	0.9, 22.5	Kvädöfjärden, Forsmark
*Terebellides stroemi*	T stroemi	*Polychaeta*	0.7	Kvädöfjärden
*Theodoxus fluviatilis*	T fluviatilis	*Gastropoda*	1.5	Forsmark

1 As used in the text and figures.

2 Average abundances (ind/m^2^) over the whole time series assessed.

The Kvädöfjärden area has an average salinity of 6–8 psu and is situated in a relatively sheltered part of the archipelago [23]. It is a reference area in the Swedish national environmental monitoring programme, and human population density as well as the level of local anthropogenic impact is hence low. The Forsmark area has an average salinity of 3–4 psu, the coastline is more exposed to the open sea compared to the Kvädöfjärden area, and is characterised by small islands and scerries. Local population density as well as the level of land use for agriculture is very low, and the sampling site represents a part of the reference area for the surveillance program of the nuclear power plant in Forsmark. As such it is located close to the power plant but not directly affected by the discharge of cooling water [24].

### Environmental variables

Data on species composition in each area were related to data representing the general environmental conditions during the same time period, at both the local (coastal, within the monitoring area) and the regional (off-shore, basin-wide) scale. Coastal zoobenthos communities are hypothesized being mainly influenced by local environmental conditions, but in this study we wanted to challenge this idea by also assessing the impact of variables acting on a basin-wide scale, see [5]. Additionally, as a proxy for large-scale climate change, on cross-basin and Baltic-wide scale, we included data on the Baltic Sea Index (BSI) [25], as large-scale climate change has been demonstrated to impact Baltic ecosystems and communities [1], [5]. Variables related to hydrological conditions were represented by data on surface water temperature, salinity, pH and oxygen, and to nutrient conditions by water transparency, nutrient concentration and nutrient load. We also included an index of the species composition of the local fish community as a proxy for changes in top-down control (Table 2 and 3, see below for further details).

**Table 2:** The variables used as predictors for the temporal development of zoobenthos communities in Kvädöfjärden.

Variable	Abbr^1^	Season	Months	Depth	Unit	Sampling station	Data prov^2^	Lag phase^3^
Local water temperature, summer	Tsu_L_	Summer	June-Aug	0–10 m	°C	Kvädöfjärden	SLU^4^	−1 year
Local water transparency^5^	*TR_L_*	Summer	Aug	NA	m	Kvädöfjärden	SLU^4^	−1 year
Local fish community composition, summer	Fsu_L_ 6	Summer	Aug	NA	NA	Kvädöfjärden	SLU^4^	−1 year
Local fish community composition, autumn	Fau_L_ 7	Autumn	Oct	NA	NA	Kvädöfjärden	SLU^4^	–1 year
Local runoff of nitrogen from land	N_L_	Annual	NA	NA	Tonnes	Östergötland county (E)	SLU^4^	–
Regional surface water temperature, spring	Tsp_R_	Spring	April	0–10 m	°C	BY15	SMHI^8^	–
Regional surface water temperature, summer	Tsu_R_	Summer	June-Aug	0–10 m	°C	BY15	SMHI^8^	−1 year
Regional surface salinity	S_R_	Annual	NA	0–10 m	psu	BY15	SMHI^8^	–
Regional surface pH	pH_R_	Annual	NA	0–10 m	NA	BY15	SMHI^8^	–
Regional surface oxygen, summer	O_R_	Summer	July-Aug	0–10 m	ml/l	BY15	SMHI^8^	−1 year
Regional surface DIN, winter	DIN_R_	Winter	Jan-Feb	0–10 m	µmol/l	BY15	SMHI^8^	–
Regional surface DIP, winter	DIP_R_	Winter	Jan-Feb	0–10 m	µmol/l	BY15	SMHI^8^	–
Baltic Sea Index, winter	BSI	Winter	Dec-March	NA	NA	NA	IFM GEOMAR^9^	–

1 Abbreviation as used in the text and figures.

2 Data provider.

3 Lag phase of the data in the DISTLM analysis.

4 Swedish University of Agricultural Sciences.

5 Excluded from analysis due to a VIF value >4 [28].

6 Explained 40% of variation in fish community structure [5].

7 Explained 45.4% of variation in fish community structure [5].

8 Swedish Meteorological and Hydrological Institute.

9 Leibniz Institute of Marine Sciences.

**Table 3:** The variables used as predictors for the temporal development of zoobenthos communities in Forsmark.

Variable	Abbr^1^	Season	Months	Depth	Unit	Sampling station	Data prov^2^	Lag phase^3^
Local water temperature, summer	Tsu_L_	Summer	Aug	0–10 m	°C	Forsmark	SLU^4^	−1 year
Local water transparency^5^	TR_L_	Summer	Aug	NA	m	Forsmark	SLU^4^	−1 year
Local fish community composition, summer	Fsu_L_ 6	Summer	Aug	NA	NA	Forsmark	SLU^4^	−1 year
Local runoff of nitrogen from land	N_L_	Annual	NA	NA	tonnes	Gävleborg county (X)	SLU^4^	–
Regional surface water temperature, spring	Tsp_R_	Spring	April-June	0–10 m	°C	SR5	SMHI^7^	–
Regional surface water temperature, summer	Tsu_R_	Summer	Aug-Sept	0–10 m	°C	SR5	SMHI^7^	−1 year
Regional surface salinity, winter	S_R_	Winter	Novr-Dec	0–10 m	psu	SR5	SMHI^7^	–
Regional surface pH	pH_R_	Annual	March, June-Sept	0–10 m	NA	SR5	SMHI^7^	–
Regional surface oxygen, winter	O_R_	Summer	Nov-Dec	0–10 m	ml/l	SR5	SMHI^7^	−1 year
Regional surface DIN, winter	DIN_R_	Winter	Nov-Dec	0–10 m	µmol/l	SR5	SMHI^7^	–
Regional surface DIP, winter	DIP_R_	Winter	Nov-Dec	0–10 m	µmol/l	SR5	SMHI^7^	–
Baltic Sea Index, Winter	BSI	Winter	Dec-March	NA	NA	NA	IFM GEOMAR^8^	–

1 Abbreviation as used in the text and figures

2 Data provider

3 Lag phase of the data in the DISTLM analysis

4 Swedish University of Agricultural Sciences.

5 Excluded from analysis due to a VIF value >4 [28].

6 Explained 37.6% of variation in fish community structure [5].

7 Swedish Meteorological and Hydrological Institute.

8 Leibniz Institute of Marine Sciences.

Local water temperature was represented by surface summer temperatures (Tsu_L_) collected in each monitoring area. Local nutrient conditions were represented by water transparency (TR_L_), as measured within each sampling area, and by nutrient load, measured as the total discharge of nitrogen from land within the county of each sampling site (N_L_; Table 2 and 3). Data on regional surface spring (Tsp_R_) and summer temperatures (Tsu_R_), salinity (S_R_), pH (pH_R_), oxygen (O_R_) and nutrient conditions (dissolved inorganic nitrogen, DIN_R_, and dissolved inorganic phosphorous, DIP_R_) was obtained from the offshore monitoring programme of the Swedish Meteorological and Hydrological Institute (SMHI; stations BY15 for Kvädöfjärden and SR5 for Forsmark, see Table 2 and 3 for further details). Since there is no monitoring of salinity levels at the zoobenthos monitoring sites, data were obtained from the closest available offshore monitoring stations for both areas. As the exchange of water between coastal and offshore areas generally is great in the Baltic, the differences in salinity from more offshore to coastal areas are typically rather small [26]. Offshore surface salinity values were hence considered to serve as an adequate proxy for salinity also in the zoobenthos monitoring sites. For oxygen concentration, similar data is not available, but the incidence of hypoxia in Kvädöfjärden and Forsmark is, however, low to non-existing (K. Mo, Department of Aquatic Resources, SLU, pers. comm.). Both areas are also rather shallow and well circulated (22–24 meters in Kvädöfjärden and at 16 meters in Forsmark). The BSI represents a somewhat regional analogue to the North Atlantic Oscillation index (NAO) [27], but directly reflects the impact of local climate variability on the oceanographic conditions over the Central Baltic Sea [25]. The index is defined as the anomalies in differences in standardized sea level pressure between Szcecin (Poland) and Oslo (Norway). Generally, positive values of the BSI conform to westerly winds over the Baltic region whereas negative values represent more easterly winds [25]. Here we used the winter (December-March) based values of BSI.

As an index of the development of the coastal fish community in each area, we used the first ordination axis from a PCO-analysis (years as samples) of local fish community composition in the same areas (see [5] for details). The sampling sites for the fish communities in both areas are situated in the vicinity of the zoobenthos monitoring sites, in Kvädöfjärden approximately one kilometre away and in Forsmark area about three kilometres. These distances are within swimming distance for the fish species used in the fish community data sets, and the sampling programs for both taxa in both areas are designed to yield representative data for the zoobenthos- and fish communities in the each area. The data represented the temporal development in species composition in August (Fsu_L_; 1971–2008 for Kvädöfjärden, and 1975–2008 for Forsmark) and October (Fau_L_, 1971–2008 for Kvädöfjärden; no data for October was available for Forsmark for the time-period assessed). The rationale for using fish community data from different seasons is that species composition of coastal fish communities in the Baltic Sea differs between seasons, as a result of species-specific differences in activity, temperature preference and migration behavior [28], [29]. Hence, the nature of a potential link between fish and zoobenthos might also differ across seasons. Generally, the fish community in the Kvädöfjärden area have been relatively stable in August during the last 40 years, but with an increase in perch [5], which is recognised as a species potentially affecting zoobenthos communities by predation on soft-bodied macro-zoobenthos as *C. volutator* [14] but to some extent also on *M. balthica* [17]. In October, however, the fish community has gone through substantial structural change in the Kvädöfjärden area with a marked decrease in zoobenthivorous species as cod, four-horned sculpin (*Triglopsis quadricornis*), whitefish (*Coregonus maraena*) and roach [5]. As for August, there has been a concurrent increase in freshwater species like perch in October. In Forsmark (August), there has been a general increase in the abundance of perch and roach and some other but less benthivorous species of fish favoured by increased water temperatures and lowered salinity levels [5]. This was mainly initiated in the early 1990's.

### Analyses

For all analyses, species with a frequency of occurrence below 5% were excluded and species data was ln(x+1) transformed to enhance normality and to reduce the influence of highly abundant species as suggested by [30]. To assess significant shifts in community composition over time, we used chronological cluster analysis [31], as implemented in Brodgar 2.5.7 (www.brodgar.com), based on the Bray-Curtis Similarity Index. This index gives a balanced weight between rare and abundant species [32], and joint absence does not contribute to similarity between samples. We chose a level of connectedness between samples of 0.5 and *α* = 0.01, in order to only include the most marked changes in each data set [32].

**Figure 2:**
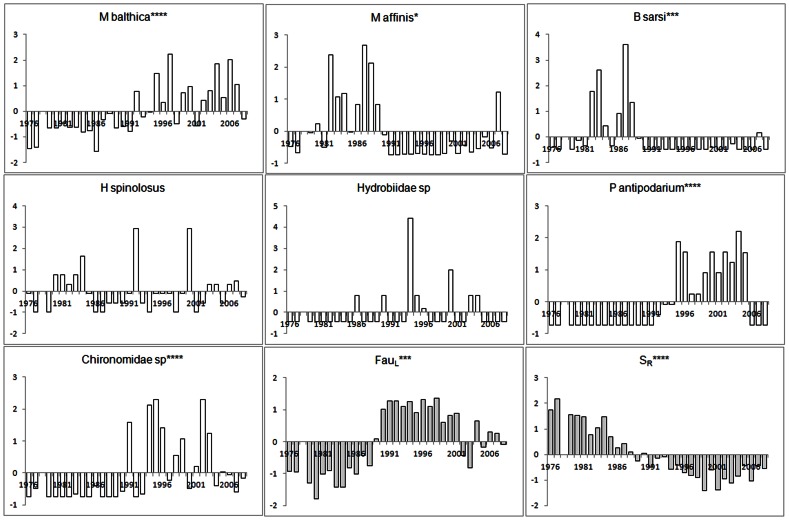
Development of species and variables contributing to temporal change community in community composition in Kvädöfjärden. The species presented (white bars) are those exhibiting a multiple metric correlation >0.2 with any of the first two ordination-axes of the PCO-analysis for each data set and the variables (grey bars) associated with community development according to the DISTLM analyses. The annual value of each species and variable is presented as the standardised deviation from the average value during the whole time-series. For abbreviations of species names and environmental variables see table 1 and 2 respectively. * denotes a significant linear trend at α = 0.05, *** α = 0.001 and **** α = 0.0001.

**Figure 3:**
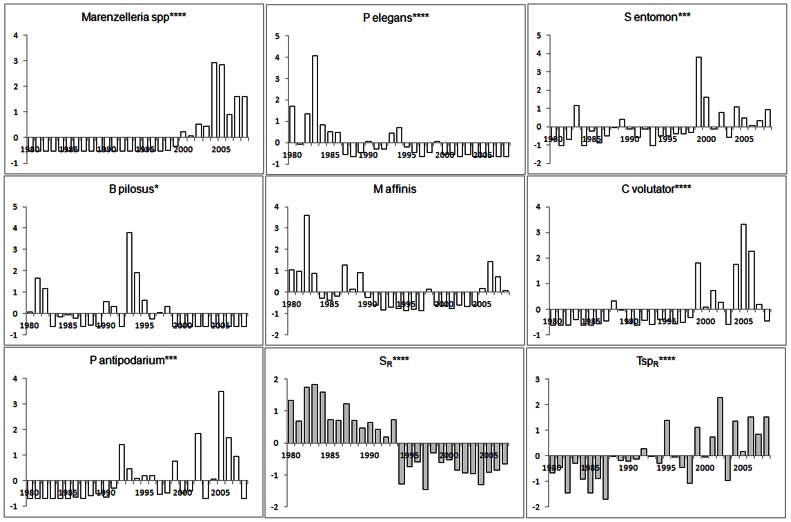
Development of species and variables contributing to temporal change community in community composition in Forsmark. The species presented (white bars) are those exhibiting a multiple metric correlation >0.2 with any of the first two ordination-axes of the PCO-analysis for each data set and the variables (grey bars) associated with community development according to the DISTLM analyses. The annual value of each species and variable is presented as the standardised deviation from the average value during the whole time-series. For abbreviations of species names and environmental variables see table 1 and 3 respectively. * denotes a significant linear trend at α = 0.05, *** α = 0.001 and **** α = 0.0001.

To assess the temporal development of the studied zoobenthos communities we further applied metric multidimensional scaling using principal coordinate analysis (PCO) [32], as implemented in PERMANOVA+ of PRIMER v6 [33]. For consistency among analyses, the PCO analyses were based on the Bray-Curtis Similarity Index and ln(x+1) transformed data. We considered species with a multiple metric correlation >0.2 with any of the first two ordination-axes as significantly contributing to the temporal development of the assessed zoobenthos community [33]. The temporal development of these species was visualised in anomaly graphs (Figure 2 and 3).

The association between zoobenthos community development and environmental variables was assessed using distance-based linear models (DISTLM), as implemented in PERMANOVA+ of PRIMER v6 [33]. DISTLM is a multivariate multiple regression routine where a resemblance matrix of a response data set is regressed against a set of explanatory variables. The resemblance matrix was based on the Bray-Curtis Similarity Index using years as samples. To reduce redundancy among the explanatory variables, we only included variables with Variation Inflation Factors (VIF) [34]≤4. Skewness of the explanatory variables was also inspected using draftman plots (pair-wise plots of all variable combinations) [35]. Local water transparency (TR_L_), was left out from modelling in both data sets as a result of redundancy with mainly fish community composition, temperatures and salinity (see Table S1, 2 and 3 for details). For some of the environmental variables a lag phase of -1 year was used since the sampling time of this data precede that of the zoobenthos community monitoring (in May) that very year (see Table 2 and 3 for details).

The variables included in the final DISTLM-models for each data set were selected using the *BEST* selection procedure in PRIMER v6 [33], based on the corrected Akaike information criterion (AICc) [36] and Bayes information criterion (BIC) [37]. As all models close to the best model (within two units) when applying the AICc criterion may be redundant, due to the inclusion of a penalty term in the expression [36], we based our final model selection on four separate steps. In the first step, the model for which the mean value of the two information criteria (AICc and BIC) was minimized was selected as the best model [33]. In the second step, the log-likelihood value for the AICc criterion of all models occurring within two units of the best model was evaluated [36], in to order account for potential influence of the penalty term in the AICc - expression. Models with a substantially higher log-likelihood value (at least two units higher than for the other models) were identified as superior. Third, the individual occurrence weights, i.e. the number of times a given variable was included in any model within two units of the best model, according to the AICc selection criterion, was calculated for each variable [36]. The variables with highest occurrence weights were identified as superior. In the fourth step, we identified variables exhibiting a significant correlation with the pattern in the species data set, using marginal F-tests as available in DISTLM (α = 0.05). Only variables identified as superior in all four steps were included in the final models for each data set. The partitioning of variation between variables in the final models was assessed using the sequential selection procedure [33]. The temporal development of the environmental variables included in the final models was visualized in anomaly graphs (Figure 2 and 3).

## Results

### Temporal development of communities

**Figure 4:**
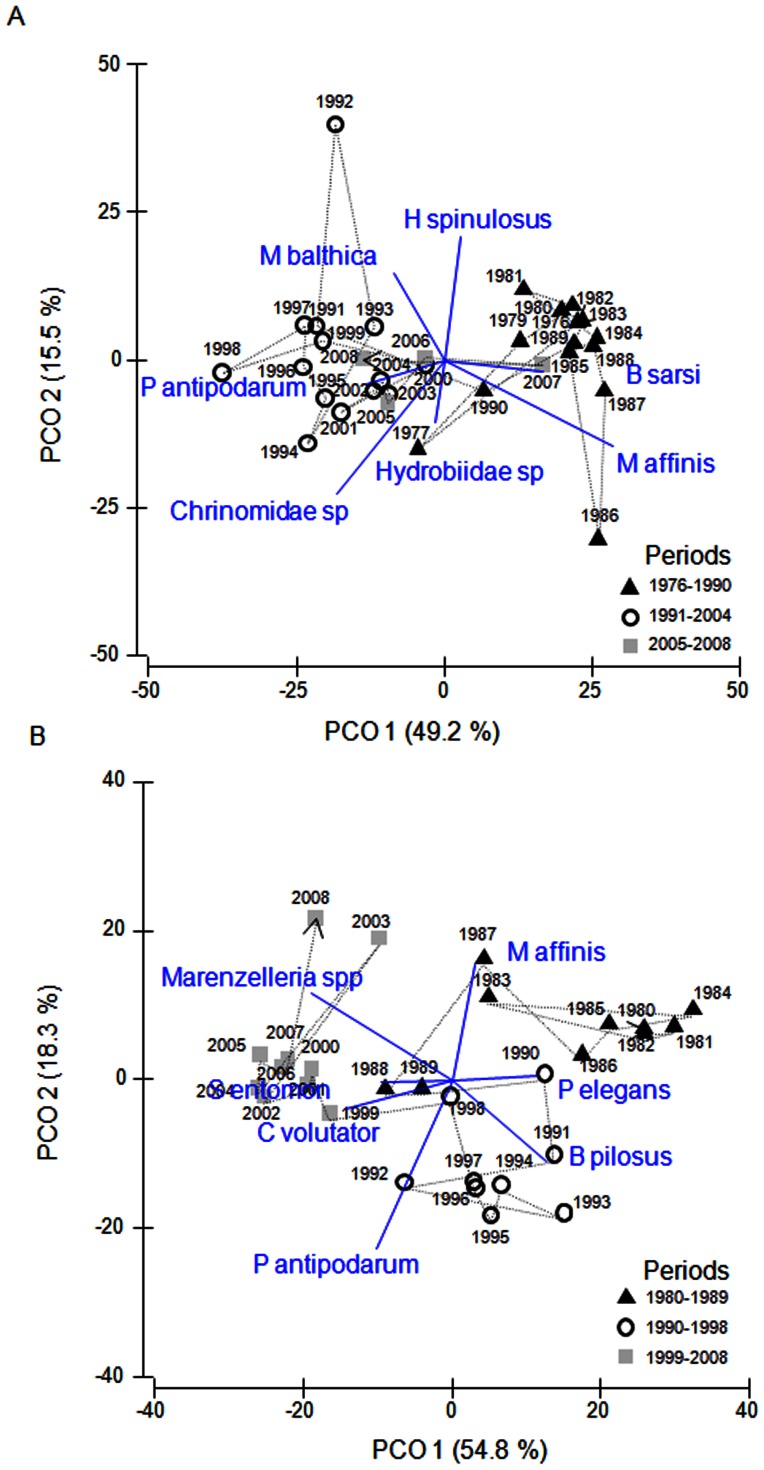
PCO-ordinations of the two zoobenthos communities assessed. Kvädöfjärden (A) and Forsmark (B). The ordinations are based on the Bray-Curtis similarity index and the projected vectors denote the abundance of species with a correlation >0.2 with any of the two first ordination axes. Years with similar species composition according to the chronological clustering analyses are indicated by the same symbols. The line indicates the temporal trajectory of the community. For abbreviation of species names, see table 2.

The first two ordination axes captured the main part of the variation in both PCO-analyses (64.7% for Kvädöfjärden, and 73.1% for Forsmark; Figure 4). According to the ordinations, the species composition of both zoobenthos communities assessed has undergone substantial changes during the last 25–30 years (Figure 4). The chronological clustering analyses suggested significant changes in community composition during the late 1980s and early 1990s in both data sets (1990/1991 in Kvädöfjärden and 1989/1990 in Forsmark). In the Forsmark data set, another significant change in species composition was suggested in 1998/1989 and in Kvädöfjärden in 2004/2005. When excluding the invasive species *Marenzelleria spp*. from the Kvädöfjärden data set, only the significant change in 1990/1991 was evident.

The zoobenthos community in the Kvädöfjärden area was characterised by high abundances of the polychaete *B. sarsi* and the amphipod *M. affinis* during the years before 1990 (Figure 2 and 4a). In the following years (1991–2008), there was substantial overlap in species composition when comparing the two different time periods identified by the chronological clustering analyses (1991–2004 and 2005–2008; Figure 4a), supporting the finding that the later change in community composition suggested by the chronological clustering analysis was dependent on the appearance of *Marenzelleria spp*. in the area. During these years, the abundance of *B. sarsi* and *M. affinis* decreased drastically with a concurrent increase in the bivalve *M. balthica*, the gastropod *P. antipodarum* and *Chrionomidae* (Figure 2). The gastropods *Hydrobia sp*. and the priapulid *H. spinolosus* also contributed to the PCO-ordination in the Kvädöfjärden data set (Figure 4a). Whereas the abundance of *H. spinolosus* peaked during the early 1990's, the abundance of *Hydrobia sp*. showed strong interannual variations but exhibited no temporal trend (Figure 2).

The development of the zoobenthos community in the Forsmark data set has followed a pattern similar to that of the Kvädöfjärden community, but exhibit an even stronger change. The three time periods of significantly different species composition suggested by the chronological clustering analysis showed little overlap in terms of dominating species (Figure 4b). Similar to the Kvädöfjärden data set, the period before the 1990s (1980–1989) was characterised by high abundances of *M. affinis* and a polychaete, *P. elegans* (Figure 3 and 4b). After 1990, the abundance of these two species decreased, and there was an increase in *P. antipodarum* and the amphipod *B. pilosus* (Figure 3 and 4b). *Macoma balthica* did not have a strong influence on the overall pattern in the Forsmark data set, but started to increase in abundance in the early 1990s, as it also did in the Kvädöfjärden area (Figure 3). During the last ten years studied (1999–2008), the zoobenthos community was characterised by above average abundances of the isopod *S. entomon* and the amphipod *C. Volutator*, but also by the invasive polychaete *Marenzelleria spp*., which appeared for the first time in 1998 (Figure 3 and 4b).

### Association with environmental variables

For both data sets, several models had AICc - and BIC - values within two units of the best model (Table 4 and 5), suggesting redundancy among models in which environmental variables that were associated to the development of the communities assessed.

**Table 4:** Outcome of the DISTLM models for the Kvädöfjärden data set.

Step1				Step 2		Step 3		Step 4
AICc		BIC		Log-likelihood			Abundance weights	Marginal test
Model		Model		Model		Variable		Pseudo-F (alfa)
**Fau_L_, Tsp_R_ & S_R_**	**203.47**	**Fau_L_, Tsp_R_ & S_R_**	**207.85**	Fau_L_, Tsp_R_ & S_R_	197.47	Fau_L_	1	1.0 (0.40)
Fau_L_, Tsp_R,_ S_R_ & Fsu_L_	203.57	Fau_L_	207.93	Fau_L_, Tsp_R,_ S_R_ & Fsu_L_	195.57	N_L_	2	0.65 (0.63)
Fau_L_, Tsp_R,_ S_R_ & N_L_	204.32	Fau_L_ & S_R,_	208.13	Fau_L_, Tsp_R,_ S_R_ & N_L_	196.32	Fau_L_	**18**	**12.9 (0.0001)**
Fau_L_, Tsp_R_, S_R_ & O_R_	204.37	Fau_L_ & Tsp_R_	208.16	Fau_L_, Tsp_R_, S_R_ & O_R_	196.37	Fsu_L_	5	1.0 (0.39)
Fau_L_, Tsp_R_, S_R_, Fsu_L_ & O_R_	204.43	Fau_L_, Tsp_R_, S_R_ & Fsu_L_	208.59	Fau_L_, Tsp_R_, S_R_, Fsu_L_ & O_R_	194.43	Tsp_R_	**13**	1.3 (0.25)
Fau_L_, Tsp_R_, S_R_, Fsu_L_ & N_L_	204.52	Fau_L_ & DIP_R_	209.16	Fau_L_, Tsp_R_, S_R_, Fsu_L_ & N_L_	194.52	Tsu_R_	1	**4.4 (0.006)**
Fau_L_ & S_R_,	204.59	Fau_L_, S_R_ & Fsu_L_	209.33	Fau_L_ & S_R_,	200.59	S_R_	**14**	**9.9 (0.0001)**
Fau_L_ & Tsp_R_	204.62	Fau_L_, Tsp_R_, S_R_ & N_L_	209.34	Fau_L_ & Tsp_R_	200.62	O_R_	3	0.82 (0.50)
Fau_L_, Tsp_R_, S_R_ & Tsu_R_	204.8	Fau_L_, Tsp_R_, S_R_ & O_R_	209.4	Fau_L_, Tsp_R_, S_R_ & Tsu_R_	196.8	pH_R_	0	**3.5 (0.017)**
Fau_L_, Tsp_R_, S_R_ & DIP_R_	204.81	Fau_L_, Tsp_R_ & N_L_	209.51	Fau_L_, Tsp_R_, S_R_ & DIP_R_	196.81	DIN_R_	0	2.5 (0.052)
Fau_L_	205.41	Fau_L_, Tsp_R_, S_R_ & Fsu_L_	209.6	**S_R_**	**203.41**	DIP_R_	4	1.5 (0.21)
Fau_L_ & DIP_R_	205.62	Fau_L_, S_R_ & O_R_	209.67	Fau_L_ & DIP_R_	201.62	BSI	0	0.95 (0.43)
Fau_L_, S_R_ & Fsu_L_	204.95	Fau_L_, Tsp_R__&_ Fsu_L_	209.73	Fau_L_, S_R_ & Fsu_L_	198.95			
Fau_L_, Tsp_R_ & N_L_	205.13	Fau_L_, Tsp_R_, TsuR & S_R_	209.82	Fau_L_, Tsp_R_ & N_L_	199.13			
Fau_L_, S_R_ & O_R_	205.29	Fau_L_, S_R_ & DIP_R_	209.83	Fau_L_, S_R_ & O_R_	199.29			
Fau_L_, Tsp_R_, S_R_ & Tsu_L_	205.29	Fau_L_, Tsp_R_ & DIP_R_	209.83	Fau_L_, Tsp_R_, S_R_ & Tsu_L_	197.29			
Fau_L_, Tsp_R__&_ Fsu_L_	205.34	Fau_L_, Tsp_R_, S_R_ & DIP_R_	209.83	Fau_L_, Tsp_R &_ Fsu_L_	199.34			
Fau_L_	205.41			**Fau_L_**	**203.41**			
Fau_L_, S_R_ & DIP_R_	205.45			Fau_L_, S_R_ & DIP_R_	199.45			
Fau_L_, Tsp_R_ & DIP_R_	205.45			Fau_L_, Tsp_R_ & DIP_R_	199.45			

**Table 5:** Outcome of the DISTLM models for the Forsmark data set.

Step1				Step 2		Step 3		Step 4
AICc		BIC		Log-likelihood			Abundance weights	Marginal test
Model		Model		Model		Variable		Pseudo-F (alfa)
**Tsp_R_ & S_R_**	**177.01**	**Tsp_R_ & S_R_**	**180.15**	Tsp_R_ & S_R_	173.01	Tsu_L_	0	1.0 (0.37)
Tsp_R_, S_R_ & DIP_R_	177.33	S_R_	180.33	Tsp_R_, S_R_ & DIP_R_	171.33	N_L_	3	0.30 (0.87)
Tsp_R_, S_R_, DIP_R_ & DIN_R_	177.59	Tsp_R_, S_R_ & DIP_R_	181.14	Tsp_R_, S_R_, DIP_R_ & DIN_R_	169.59	Fsu_L_	0	**4.7 (0.006)**
S_R_	178.06	S_R_ & DIP_R_	181.24	**S_R_**	**176.06**	Tsp_R_	**9**	**10.6 (0.0001)**
S_R_ & DIP_R_	178.10	S_R_ & O_R_	181.65	S_R_ & DIP_R_	174.1	Tsu_R_	1	**2.9 (0.041)**
Tsp_R_, S_R_ & N_L_	178.16	Tsp_R_, S_R_, DIP_R_ & DIN_R_	181.82	Tsp_R_, S_R_ & N_L_	172.16	S_R_	**14**	**12.8 (0.0001)**
Tsp_R_, S_R_ & Tsu_R_	178.16	Tsp_R_	181.93	Tsp_R_, S_R_ & Tsu_R_	172.16	O_R_	2	1.5 (0.19)
S_R_, DIP_R_ & DIN_R_	178.35	Tsp_R_, S_R_ & N_L_	181.97	S_R_, DIP_R_ & DIN_R_	172.35	pH_R_	1	1.1 (0.33)
Tsp_R_, S_R_, N_L_ & DIP_R_	178.47	Tsp_R_, S_R_ & Tsu_R_	181.97	Tsp_R_, S_R_, N_L_ & DIP_R_	170.47	DIN_R_	4	0.39 (0.79)
S_R_ & O_R_	178.51	S_R_ & pH_R_	182.10	S_R_ & O_R_	174.51	DIP_R_	6	2.8 (0.049)
Tsp_R_, S_R_ & O_R_	178.51	S_R_, DIP_R_ & DIN_R_	182.15	Tsp_R_, S_R_ & O_R_	172.51	BSI	0	0.84 (0.47)
Tsp_R_, S_R_, N_L_, DIN_R_ & DIP_R_	178.75			Tsp_R_, S_R_, N_L_, DIN_R_ & DIP_R_	168.75			
S_R_ & pH_R_	178.96			S_R_ & pH_R_	174.96			
Tsp_R_, S_R_, & DIN_R_	179.01			Tsp_R_, S_R_, & DIN_R_	173.01			

Bold values indicate superior models (selection step 1 and 2) and variables (selection steps3 and 4).

For the Kvädöfjärden data set, in total 20 (AICc) and 17 (BIC) models were found within two units of the best model (Table 4). According to both the AICc and BIC criteria, however, the model with the lowest values included fish community structure in autumn (Fau_L_), offshore spring temperature (Tsp_R_) and offshore salinity (S_R_). With respect to log-likelihood values for the AICc criterion, the two models including only Fau_L_ and only Tsp_R_ were identified as superior (Table 4). With respect to the AICc abundance weights, Fau_L_ occurred in 18, S_R_ in 14 and Tsp_R_ in 13 of the 20 highest ranked models (Table 4). No other variable occurred in more than five of the highest ranked 20 models. Of the three variables, only Fau_L_ and S_R_ exhibited a significant correlation with the observed pattern in the zoobenthos data set (Marginal tests, Table 4). The correlation with offshore summer temperature (Tsu_R_) and pH (pH_R_) was, however, also significant, but both these variables had substantially lower Pseudo-F values, low abundance weights (one and zero, respectively) and were not included in any of the superior models in step 1 and 2 of the selection procedure (Table 4). In all, the combined model selection procedure indicated that the development of the zoobenthos community in the Kvädöfjärden area could primarily be associated with local fish community structure in autumn and offshore salinity. Together, the model including these variables explained 36.9% (Fau_L_ 30.0% and S_R_ 6.9%, respectively) of the total variation assessed.

For the Forsmark data set, the degree of redundancy among models appeared to be somewhat lower. In total, 14 (AICc) and 11 (BIC) models occurred within two units of the best model (Table 5). Similar to the Kvädöfjärden data set, however, the model including both Tsp_R_ and S_R_ had the lowest values for both information criteria. The model including S_R_ only had the overall highest log-likelihood value (more than three units higher than the model including Tsp_R_ and S_R_; Table 5). S_R_ had the overall highest abundance weight (14), and was included in all the highest ranked models according to the AICc criterion. The abundance weight for TspR was nine. Both variables were, however, significantly correlated to the development of zoobenthos species composition, according to the marginal tests (Table 5). Three additional variables, fish community composition in August (Fsu_L_), Tsu_R_, and regional dissolved inorganic phosphorous (DIP_R_) were also identified a significant in the marginal tests, but were not considered further as they did not comply with the criteria of the other selection steps. Local fish community composition in the autumn, which was included in the final model of the Kvädöfjärden data set, was not assessed in the analyses of the Forsmark data set, as data was only available for years between 1980 and 2002. However, when the time series for all variables were shortened to only include these years, and the analyses were re-run, for comparison, Fau_L_ was included in the final model also for the Forsmark data set (results not shown). Due to substantial co-linearity between Fau_L_ and S_R_ (*R = *0.83) in this analysis, the temporal development of S_R_ in the analysis considering the whole time-period assessed (1980–2008) might to some extent also reflect changes in the local fish community in autumn in the area (see also [5]). In all, offshore salinity was identified as the variable mainly associated with the development of the zoobenthos community in the Forsmark area. S_R_ explained 32.1% of the total variation assessed. When also including offshore spring temperature (Tsp_R_), the amount of variation explained increased to 39.9%.

Over the time-periods assessed, there has been a decrease in offshore salinity and increase in spring temperatures in both areas during the last 25–30 years (Figure 2 and 3). In the Baltic Proper (including Kvädöfjärden), regional spring temperature during the first five years assessed (1976–1981) was on average 4.4°C, as compared to 7.8°C in the last five years (2004–2008). Corresponding figures for salinity were 7.7 and 7.1, respectively. During the assessed time-period, regional spring temperature increased in the Bothnian Sea (including Forsmark), from on average 3.6°C (1980–1984) to 7.7°C (2004–2008), and salinity decreased from on average 6.0 to 5.4 in the same years. For the temporal development of local fish communities, see the Methods section. With respect to the other environmental variables considered in the DISTLM analysis but not included in the final models, there was a significant linear increase in DIP and regional summer temperatures in both basins, and in coastal summer temperature in the Forsmark area.

## Discussion

In this study we show that coastal zoobenthos communities in two Baltic Sea areas have gone through substantial changes in species composition over time during the last 30 years, and that these changes to some extent may be associated with concurrent changes in local fish communities and changes in salinity levels and water temperature. The findings suggest that the temporal development of coastal zoobenthos communities in the areas is not only driven by local factors, but also by common large scale pressure variables.

### Temporal development of communities

We found evidence for substantial turn-over in species composition in both zoobenthos communities assessed. Given some apparent differences in species composition, as well as in the physical and hydrographical settings between the areas, some community-specific responses in development may be expected. In both areas, however, there were common patterns with respect to both the temporal development of certain taxa and to the timing of significant changes in community composition.

The decrease in marine polychaetes in both communities has also been observed in other studies. Data from deeper parts of the Baltic Proper and Gulf of Finland show that the abundance of *B. sarsi* has decreased since the 1980's [11], [38]. To the best of our knowledge, our study is the first to describe the long-term development of *P. elegans* (Forsmark data set) in the Baltic Sea. Polychaetes are important food for fish, including *B. sarsi*, which is a main prey of cod [39]. The observed decrease of the amphipod *M. affinis* in the early 1990's in both communities also corroborates previous documentations throughout the Baltic Sea [11], [13], [40]. Being a glacial relict, *M. affinis* is sensitive to increasing temperatures [41], but also to hazardous substances, hypoxia and eutrophication [13], reviewed in [40]. Interestingly, an increase in the abundance of *M. affinis* was observed during the last few years of study in both data sets, which might signal improved environmental status in the areas [40].

Concurrent with the decline in the species discussed above, the gastropod *P. antipodarum* and bivalve *M. balthica* exhibited increases in abundance in both communities. *Potamopyrgus antipodarum* is originally an introduced species, although it colonised the Baltic Sea more than a century ago [42], and is now important part of the diet of fish, for example for roach [43]. Thus, the increase of the species observed in this study cannot be explained by recent introduction to the Baltic, but may be related to increased ecological opportunity following the collapse of *M. affinis* in combination with its wide temperature and salinity tolerance, high reproductive capacity, and tolerance to anthropogenic disturbance [44]. Interestingly, in parallel with increasing abundances of *M. affinis* during the last few years in both areas, the abundance of *P. antipodarum* were again below average. Increases in *M. balthica*, as observed in both communities in this study, were also observed in the Gulf of Finland during the early 2000's [11], [13]. *Macoma balthica* is the most common bivalve in the Baltic, and is an important prey for roach, flounder and to some extent also perch [17], [43]. The species is considered highly tolerant to environmental perturbations [45], and the rationale for the recent increase of the species might hence be similar to that for *P. antipodarum*.

In addition to the common patterns observed for both communities, an increase in chironomids was observed in the Kvädöfjärden data set, and in *S. entomon, Marenzelleria spp*. and *C. volutator* in the Forsmark data set. We know of no other studies of the long-term development of chironomids and *C. volutator* in the Baltic Sea, but chironomids are known to be tolerant to changes in temperature, hypoxia, increased levels of nutrients and lowered salinity [46], [47], and *C. volutator* being a salinity tolerant species [48]. *Marenzelleria spp*. is an invasive species introduced to the Baltic in the mid 1990's [42], and the species is now considered established in the northern Baltic Sea [49]. *Saduria entomon* is an important species in the coastal as well as the open sea benthic ecosystem, both as a predator on other zoobenthic species such as *M. affinis* [50], [51], and as key prey for fish, such as cod and four-horn sculpin (*Triglopsis quadricornis*) (reviewed in [52]). It is also rather resistant to abiotic stress, reviewed in [53]. Interestingly, the increase of *S. entomon* in the Forsmark data set concurs with the decline in *M. affinis*, cod and four-horn sculpin in the same area [5].

Besides showing different relationships to changes in environmental variables, the species characterizing different time-periods in both communities assessed also have different ecological roles and functions. The zoobenthos community in the Kvädöfjärden area has changed from a state with relatively high biomass of detritus feeders (*B. sarsi* and *M. affinis*), plankton feeders (*M. affinis*) and predatory zoobenthos species (*B. sarsi* and *H. Spinolosus*; mid 1970s to the early 1990s), to a state characterized by increasing abundances of filtration and suspension feeders (*M. balthica*) and grazers (*P. Antipodarum* ¸ between 1991–2008). During the later years the abundance of detritus feeders and deposit feeders (*Marenzelleria spp*. and *Chrionomidae*) has, however, increased again. In the Forsmark area a similar transition was seen. The years before the early 1990s was characterised by relative high abundances of detritus and plankton feeders (*P. elegans* and *M. affinis*), followed by a state with increasing abundances of grazers (*P. antipodarum* and *B. pilosus*) and filtration and suspension feeders (*M. balthica*). During the last ten years assessed, the abundances of predators and scavengers (*S. entomon*), detritus and deposit feeders (*Marenzelleria spp*. and *C. Volutator*) were relatively high in the Forsmark area.

### Relation to environmental variables

What variables were then associated with the changes in species composition in this study? We observed quite some redundancy among competing models for both data sets, but some general patterns were discernible. For both communities offshore salinity were included in the final models. In the Kvädöfjärden data set, however, local fish community structure in autumn was the key contributing variable, and in the Forsmark data set offshore spring surface temperature was also included in the final models. When shortening the time-series for the Forsmark data set, in order to include available data for coastal fish community in the autumn (see [5]), an association between this variable and the development of the zoobenthos community in the area was observed. Offshore spring temperature fulfilled all but one of the model selection criteria (i.e. significance in marginal tests) in the Kvädöfjärden data set, hence indicating an association to water temperature also in this area. We hence conclude that climate and fish community structure are the main factors associated with the temporal development of the assessed zoobenthos communities.

These findings are generally supported by the ecological characteristics of the species mainly contributing to the temporal development of the communities assessed. For example, a long-term decrease in salinity might explain the observed decline in the marine polychaetes (*B. sarsi* and *P. elegans*), which are sensitive to low salinity levels, reviewed in [54], and the increase in species tolerant to changes salinity, like chironomids, *P. antipodarum*, and *C. volutator* [44], [46]–[48]. Similarly, increased water temperature might contribute to the decrease in cold-water species like *M. affinis* [41]. The nutrient related variables did not have an overall strong contribution to the observed changes in zoobenthos in our study. Other studies have, however, shown that the structure of zoobenthos communities exhibit a general response to hydrography [8], [11], [13], and are sensitive to changes in salinity, temperature, oxygen conditions and eutrophication [6]. Moreover, the decline in zoobenthos communities observed in deeper areas of the Baltic Sea during the mid 1990's was mainly linked to a decrease in salinity and oxygen levels [11]. As a contrast, [55] found that local nutrient loads explained variation in abundance and biomass of zoobenthos in a shallow coastal area (Archipelago Sea) since the early 1970s. In contrast to our study, [55] focused on changes in total abundance and biomass of the communities assessed rather than changes in species composition, which may explain the different results of [55] compared to this study. Moreover, however, the Archipelago Sea is more eutrophied than the areas included in our study [56], which may further explain the stronger relationship between nutrient load and biota in [55].

The observed association to fish community structure in this study may result from both predation effects from fish on zoobenthos, as well as from a common response to large scale environmental change. Fish predation has been shown to affect zoobenthos community composition in earlier studies [14], [20], [21], and top-down related effects over time in our study might at least partly be explained by the decreases in important zoobenthivorous fish species like cod, four-horned sculpin, whitefish and roach in the Kvädöfjärden area [5], causing a predatory release of their zoobenthic prey. The recent increase in *S. entomon*, for example, coincides with the decrease in cod and four-horn sculpin in the area [5]. Similarly, the increase in molluscs in both areas coincides with the long-term decrease in roach [5], which feed predominantly on molluscs [14], [43]. A top-down effect is further supported by the timing of change in zoobenthos- and fish community structure in both areas. Significant changes in fish community structure typically occurred a few years before those observed for zoobenthos [5]. In both Kvädöfjärden and Forsmark changes in fish community structure occurred in 1988/1989, but for zoobenthos the changes occurred in 1990/1991 (Kvädöfjärden) and 1989/1990 (Forsmark). Due to the generally shorter generation times in zoobenthos species compared to fish, a response in zoobenthos community structure to environmental change would be expected to happen before, not after, a change in the fish community, if both communities exhibit parallel responses to changes in environmental conditions. In contrast to these patterns, however, we observed simultaneous increases for *C. volutator* and its predator perch, as well as for *B. sarsi* and its main predator cod, suggesting that both predators and prey in these cases are favored by similar environmental conditions. Hence, some indications of environmental forcing in shaping both zoobenthos- and fish communities were also present in some cases, suggesting that both top-down regulation and climate forcing influence the structure of coastal zoobenthos communities in the studied areas. Whereas predation effects from fish might be important in explaining the development of certain species, reviewed in [19], other parts of the zoobenthos communities assessed might have responded more strongly to climate induced effects. Although an association with the autumn fish community was observed, we found no significant link between zoobenthos community structure and warm-water fish community composition in summer. This might reflect the more pronounced changes- in species composition observed in the cold-water fish community than in the warm-water fish community, especially concerning key zoobenthivorous fish species [5]. This pattern suggests that top-down regulation of coastal zoobenthos communities may have a direct link to fisheries management, through the regulation of fishing pressure on some influential zoobenthivorous fish species [57]. It might, however, also be related to effects of changes in salinity and temperature on the structure of fish communities, and hence indirectly on the nature of the predation pressure [5].

The amount of variation explained by the final models was typically rather low, between 30 and 40%. This is likely explained by the fact that only a sub set of potentially important variables affecting coastal zoobenthos communities was assessed in this study. Due to for example restrictions in the availability of data, we have chosen to focus on the potential effects of mainly environmental pressure variables in this study, hence omitting those related to inter- and intraspecific interactions within the zoobenthos communities as well as the effects of hazardous substances and hypoxia. Predation from the isopod *S. entomon* has been shown to have a strong structuring role on zoobenthos communities in the northern Baltic Sea [51], [58], and the collapse of *M. affinis* in the early 1990s might, for example, also have increased the ecological niche for opportunistic species like *P. antipodarum*. Moreover, the effects of hypoxia in the areas in focus in this study is probably limited, as they are rather shallow and well circulated (22–24 meters in Kvädöfjärden and at 16 meters in Forsmark), and as such hypoxia is not regularly observed here (K. Mo, Department of Aquatic Resources, SLU, pers. comm.).

The results presented in this study provide insights for a deeper understanding of the association between long-term change in environmental variables and the composition of coastal zoobenthos communities. Our findings suggest that coastal zoobenthos communities along the Swedish coast have changed substantially with respect to structure and function during the last 30 years. Generally, an increase in grazers as well as in suspension and deposit feeders over time may be related both to climate forcing and top-down regulation from fish. We therefore advocate that future status assessments of zoobenthos communities in the Balic Sea should consider potential effects from both climate and food-web interactions in light of an ecosystem-based approach.

## Supporting Information

Table S1 **Cross-correlation matrix (r-values) for the variables used as predictors for the temporal development of zoobenthos communities.**
(DOCX)
