# Supplementary material for: Top-Down Regulation, Climate and Multi-Decadal Changes in Coastal Zoobenthos Communities in Two Baltic Sea Areas
Source: PLoS One. 2013 May 24;8(5):e64767. doi: 10.1371/journal.pone.0064767 (PMC3663797; doi:10.1371/journal.pone.0064767)
Supplement: Table S1 — Cross-correlation matrix (r-values) for the variables used as predictors for the temporal development of zoobenthos communities. (DOCX) [file pone.0064767.s001.docx]

**Table S1.**

Cross-correlation matrix (r-values) for the variables used as predictors for the temporal development of zoobenthos communities.

| **Data set** | **Scale** | **Variable** | Tsu_L_ | TR_L_^1^ | N_L_ | Fau_L_ | Fsu_L_ | Tsp_R_ | Tsu_R_ | S_R_ | O_R_ | pH_R_ | DIN_R_ | DIP_R_ |
| --- | --- | --- | --- | --- | --- | --- | --- | --- | --- | --- | --- | --- | --- | --- |
| Kvädöfjärden | Local | Tsu_L_ |  |  |  |  |  |  |  |  |  |  |  |  |
|  |  | TR_L_^1^ | -0.26 |  |  |  |  |  |  |  |  |  |  |  |
|  |  | N_L_ | 0.04 | -0.20 |  |  |  |  |  |  |  |  |  |  |
|  |  | Fau_L_ | 0.14 | -0.42 | -0.08 |  |  |  |  |  |  |  |  |  |
|  |  | Fsu_L_ | -0.27 | -0.01 | 0.01 | 0.05 |  |  |  |  |  |  |  |  |
|  | Regional | Tsp_R_ | -0.06 | -0.35 | 0.22 | 0.21 | 0.06 |  |  |  |  |  |  |  |
|  |  | Tsu_R_ | 0.41 | -0.30 | -0.10 | 0.45 | -0.14 | 0.30 |  |  |  |  |  |  |
|  |  | S_R_ | -0.06 | 0.73 | -0.06 | -0.72 | -0.32 | -0.32 | -0.44 |  |  |  |  |  |
|  |  | O_R_ | 0.24 | -0.15 | 0.21 | 0.04 | -0.43 | -0.12 | -0.01 | 0.14 |  |  |  |  |
|  |  | pH_R_ | -0.37 | 0.08 | 0.18 | -0.40 | 0.32 | 0.05 | -0.50 | 0.30 | -0.13 |  |  |  |
|  |  | DIN_R_ | -0.17 | 0.06 | -0.09 | 0.47 | 0.39 | -0.07 | 0.23 | -0.32 | -0.23 | 0.05 |  |  |
|  |  | DIP_R_ | 0.10 | -0.22 | -0.20 | 0.01 | 0.18 | 0.20 | 0.19 | -0.19 | -0.10 | 0.07 | 0.02 |  |
|  | Global | BSI | 0.08 | -0.02 | -0.36 | 0.38 | 0.10 | 0.39 | 0.46 | -0.26 | -0.34 | -0.38 | 0.21 | 0.14 |
|  |  |  |  |  |  |  |  |  |  |  |  |  |  |  |
| Forsmark | Local | Tsu_L_ |  |  |  |  |  |  |  |  |  |  |  |  |
|  |  | TR_L_^1^ | -0.29 |  |  |  |  |  |  |  |  |  |  |  |
|  |  | N_L_ | -0.11 | 0.30 |  |  |  |  |  |  |  |  |  |  |
|  |  | Fau_L_ | - | - | - |  |  |  |  |  |  |  |  |  |
|  |  | Fsu_L_ | -0.56 | 0.48 | -0.01 | - |  |  |  |  |  |  |  |  |
|  | Regional | Tsp_R_ | 0.15 | -0.42 | -0.15 | - | -0.31 |  |  |  |  |  |  |  |
|  |  | Tsu_R_ | 0.53 | -0.12 | -0.13 | - | -0.50 | 0.18 |  |  |  |  |  |  |
|  |  | S_R_ | -0.39 | 0.66 | 0.17 | - | 0.62 | -0.61 | -0.44 |  |  |  |  |  |
|  |  | O_R_ | 0.00 | -0.07 | -0.18 | - | -0.15 | -0.32 | 0.02 | 0.04 |  |  |  |  |
|  |  | pH_R_ | -0.09 | 0.01 | 0.09 | - | 0.09 | 0.44 | 0.03 | -0.02 | -0.10 |  |  |  |
|  |  | DIN_R_ | -0.07 | -0.20 | -0.02 | - | -0.21 | 0.01 | -0.11 | 0.03 | 0.15 | 0.30 |  |  |
|  |  | DIP_R_ | 0.01 | -0.06 | -0.14 | - | -0.30 | 0.29 | 0.21 | -0.22 | -0.18 | 0.23 | 0.52 |  |
|  | Global | BSI | -0.07 | 0.05 | 0.25 | - | -0.21 | 0.18 | 0.11 | -0.06 | -0.08 | 0.06 | 0.07 | -0.07 |

^1^ Redundancy (VIF > 4) and exclusion from further analyses [28]
